# The olfactory route is a potential way for SARS-CoV-2 to invade the central nervous system of rhesus monkeys

**DOI:** 10.1038/s41392-021-00591-7

**Published:** 2021-04-24

**Authors:** Li Jiao, Yun Yang, Wenhai Yu, Yuan Zhao, Haiting Long, Jiahong Gao, Kaiyun Ding, Chunxia Ma, Jingmei Li, Siwen Zhao, Haixuan Wang, Haiyan Li, Mengli Yang, Jingwen Xu, Junbin Wang, Jing Yang, Dexuan Kuang, Fangyu Luo, Xingli Qian, Longjiang Xu, Bin Yin, Wei Liu, Hongqi Liu, Shuaiyao Lu, Xiaozhong Peng

**Affiliations:** 1 National Kunming High-Level Biosafety Primate Research Center, Institute of Medical Biology, Chinese Academy of Medical Sciences and Peking Union Medical College, Kunming, Yunnan China; 2 State Key Laboratory of Medical Molecular Biology, Department of Molecular Biology and Biochemistry, Institute of Basic Medical Sciences, Medical Primate Research Center, Neuroscience Center, Chinese Academy of Medical Sciences, School of Basic Medicine Peking Union Medical College, Beijing, China; 3 Department of Anatomy, Institute of Basic Medical Sciences, Chinese Academy of Medical Sciences, School of Basic Medicine Peking Union Medical College, Beijing, China

**Keywords:** Infection, Diseases of the nervous system

## Abstract

Neurological manifestations are frequently reported in the COVID-19 patients. Neuromechanism of SARS-CoV-2 remains to be elucidated. In this study, we explored the mechanisms of SARS-CoV-2 neurotropism via our established non-human primate model of COVID-19. In rhesus monkey, SARS-CoV-2 invades the CNS primarily via the olfactory bulb. Thereafter, viruses rapidly spread to functional areas of the central nervous system, such as hippocampus, thalamus, and medulla oblongata. The infection of SARS-CoV-2 induces the inflammation possibly by targeting neurons, microglia, and astrocytes in the CNS. Consistently, SARS-CoV-2 infects neuro-derived SK-N-SH, glial-derived U251, and brain microvascular endothelial cells in vitro. To our knowledge, this is the first experimental evidence of SARS-CoV-2 neuroinvasion in the NHP model, which provides important insights into the CNS-related pathogenesis of SARS-CoV-2.

## Introduction

Severe acute respiratory syndrome coronavirus 2 (SARS-CoV-2) was first reported in December 2019^1^ and have caused the global pandemic of the acute and highly contagious coronavirus disease 2019 (COVID-19).^2^ In the COVID-19 patients, SARS-CoV-2 infection is accompanied by neurological complications, such as encephalitis^3^ and acute polyradiculitis.^4^ Additionally, some COVID-19 patients show neurologic signs, such as headache, dizziness, nausea, anosmia, and ageusia.^5^ The duration of insomnia symptoms is longer than that of fever,^6^ suggesting that SARS-CoV-2 infection could lead to neurological damages. SARS-CoV-2 viral particles have been found in the frontal lobe and cerebrospinal fluid (CSF) of COVID-19 patients,^3,7^ and SARS-CoV-2 replication can be detectable in the brains of human angiotensin-converting enzyme 2 (hACE2) knock-in mice.^8–10^ These evidences implicate that SARS-CoV-2 potentially invades the central nervous system (CNS) of COVID-19 patients and causes the neurological complications.

SARS-CoV-2 belongs to the same β coronavirus (β-CoV) genus as SARS-CoV first reported in 2003, and they share approximately 80% genomic homology.^11^ Most β-CoVs have a neuroinvasive propensity.^12,13^ SARS-CoV-2 enters host cells using the receptor ACE2 and transmembrane serine protease 2 (TMPRSS2) as same as SARS-CoV.^14,15^ These two key factors for SARS-CoV-2 infection are highly expressed in nasal goblet cells and ciliary cells.^16^ In both symptomatic and asymptomatic COVID-19 patients, nasal swabs have yielded higher viral loads than throat swabs.^11^ These results indicate that the nasal epithelium could be the portal site of the initial SARS-CoV-2 infection. The known neurotropic viruses generally enter the CNS through olfactory neurons, with subsequent transneuronal spread to other sites of the CNS. Intranasally^17^ or intracranially^18^ inoculated SARS-CoV has been detected throughout the brains of hACE2 transgenic mice, implying that SARS-CoV-2 may use olfactory neurons as the major access to the CNS. In addition, brains from autopsied COVID-19 patients showed SARS-CoV-2 invasion and replication in both the olfactory bulb and hypothalamus,^7,19,20^ indicating that SARS-CoV-2 may invade to CNS via the olfactory route in some individuals. Such an entry route was proved to be possible in K18-hACE2 mice following intranasal inoculation of SARS-CoV-2.^10^ Due to the limitation of CNS specimens of COVID-19 patients, it is difficult to fully elucidate SARS-CoV-2-induced neurological diseases.

Non-human primate (NHP) models can recapitulate several aspects of human disease, which is crucial to understand the pathogenic processes involved in SARS-CoV-2 infection and the development of vaccines and antivirals. It was reported that intranasal inoculation with SARS-CoV-2 results in an acute lung injury in NHPs, associated with high levels of inflammatory cytokines and accumulation of immune cells in the lung.^21–23^ These models provide an effective means to study the neurological complications associated with SARS-CoV-2 infection. However, there is limited information about the neurotropism of SARS-CoV-2 and replication ability in the CNS.

SARS-CoV-2 neurotropism, the route of invading the CNS, and the distribution of the virus are essential for the diagnosis, prognosis, and intervention of COVID-19. Therefore, in this study we used the established rhesus monkey model of COVID-19 to further address the invasion of SARS-CoV-2 to the CNS, including the possible route and the pathogenesis. We showed that SARS-CoV-2 RNA was detectable in the nasal mucosa, olfactory trigone, and the entorhinal area in chronological order. The infection of SARS-CoV-2 induced the inflammation possibly by targeting neurons, microglia, and astrocytes in the CNS in vivo and in vitro. The data suggested that SARS-CoV-2 could be trafficked to the brain possibility via the olfactory route and caused the upregulation of neuroinflammatory factors, as reported by K18-hACE2 mice model.^10^ To our knowledge, this is the first experimental evidence of SARS-CoV-2 neuroinvasion in the NHP model, which provides important insights into the CNS-related pathogenesis of SARS-CoV-2.

## Results

### Neuroinvasion of SARS-CoV-2 to the CNS via the olfactory route in rhesus monkeys

**Fig. 1:**
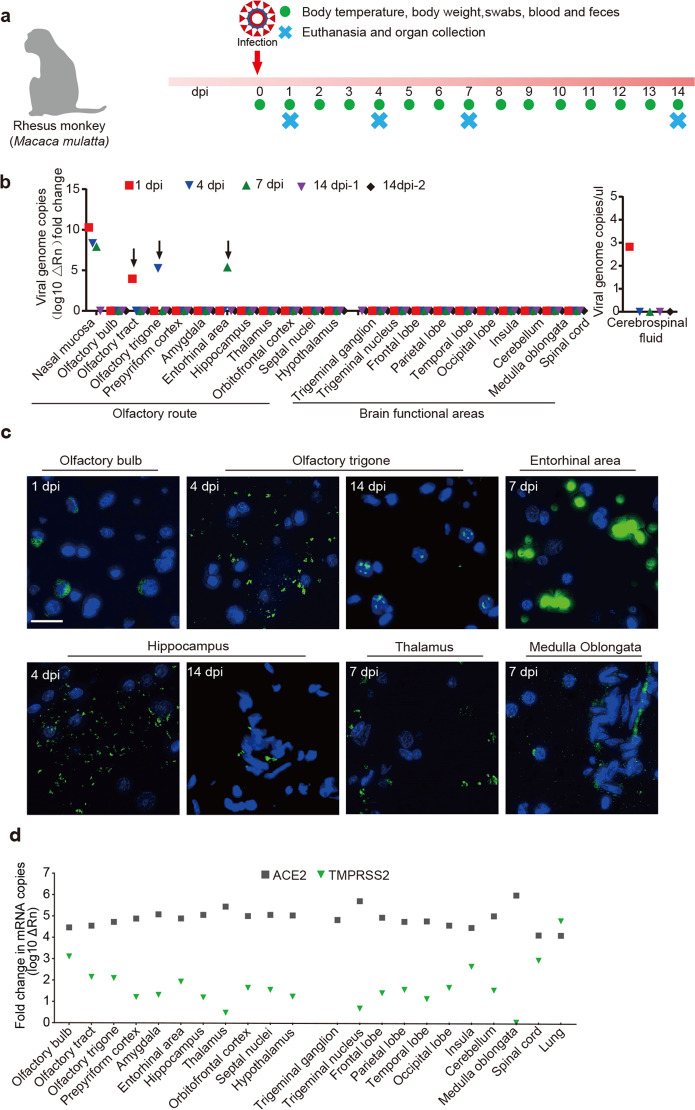
SARS-CoV-2 invades the CNS in rhesus monkeys post intranasal inoculation. **a** Scheme of the experimental design of intranasal SARS-CoV-2 inoculation. Five rhesus monkeys (3–5 years old) were intranasally inoculated with 1 × 10^7^ PFU of SARS-CoV-2 in 1 mL PBS. Monkeys were observed for clinical signs, including body temperature, body weight, sample collection, chest radiograph, and necropsies as indicated. **b** Viral load in CNS tissues from SARS-CoV-2-inoculated rhesus macaques. On 1, 4, 7, and 14 dpi, animals were necropsied and tissue samples in the CNS were collected. Viral genomic RNA was quantitated by quantitative real-time polymerase chain reaction (qRT-PCR). **c** Immunofluorescence (IF) staining of viral N protein (green) in CNS tissues from rhesus macaques infected with SARS-CoV-2. The sections were counterstained with DAPI for nuclei. Each data point represents an independent field of view from slides subjected to IF. Scale bar, 50 μm. **d** qRT-PCR analysis of angiotensin-converting enzyme 2 (ACE2) and TMPRSS2 mRNA expression in the brain tissues from the uninfected rhesus macaque

Our recently established NHP model of COVID-19 was slightly modified and then used in this study of SARS-CoV-2 neuroinvasion.^22^ Five rhesus monkeys (4–5 years of age; Supplementary Table S1) were intranasally inoculated with SARS-CoV-2, followed by the indicated sampling and analysis (Fig. 1a). No significant change of overt clinical signs (data not shown) or respiration (Supplementary Fig. S1a) was observed in any of the infected animals post intranasal viral inoculation. The viral genomic RNA in the blood was detectable in some of the animals (Supplementary Fig. S1b).

To determine whether SARS-CoV-2 can be transported into the CNS following intranasal inoculation, these five rhesus monkeys were sacrificed on days 1 (1), 4 (1), 7 (1) and 14 (2) post inoculation (dpi), respectively (Fig. 1a). Then viral loads in the nasal mucosa and CNS samples were analyzed by quantitative real-time polymerase chain reaction (qRT-PCR). From 1 to 7 dpi, SARS-CoV-2 RNA was detectable in the nasal mucosa, followed by viral appearance in the CSF, the olfactory trigone, and the entorhinal area on 1, 4, or 7 dpi, respectively (Fig. 1b), suggesting that SARS-CoV-2 transports from the nasal mucosa to the CNS via the olfactory route post intranasal inoculation. Immunofluorescence (IF) staining showed the viral antigen nucleoprotein (NP) in the olfactory bulb on 1 dpi. After 3 days (on 4 dpi), the NP distributed more widely to olfactory trigone and hippocampus, then appeared in the entorhinal area, thalamus, and medulla oblongata on 7 dpi and finally prepyriform cortex and hippocampus on 14 dpi (Fig. 1c). ACE2, the important receptors for SARS-CoV-2 infection, was highly expressed in the olfactory bulb, olfactory trigone, entorhinal area, hippocampus, and thalamus but the other important factor TMPRSS2 with the relatively lower level of expression (Fig. 1d and Supplementary Fig. S1c). These data suggest that SARS-CoV-2 invades the CNS via the olfactory route after intranasal inoculation.

### Neuroinflammation induced in the CNS by SARS-CoV-2 infection post intranasal inoculation

**Fig. 2:**
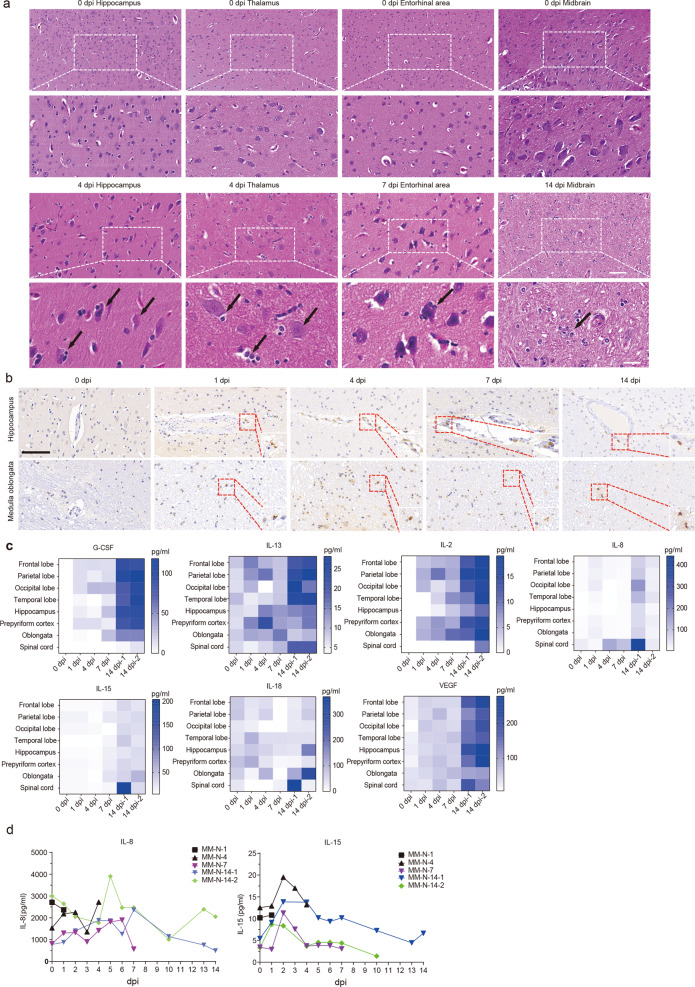
SARS-CoV-2 invasion results in inflammation and pathological changes in the CNS. **a** Hematoxylin and eosin (H&E) staining showed that intranasal SARS-CoV-2 inoculation caused different degrees of neuronal death, glial hyperplasia, and edema and perivascular inflammatory cell infiltration in the brain compared with PBS control (0 dpi), which is described in the text. Scale bar (upper panel), 50 μm; scale bar (lower panel), 20 μm.(b) Immunochemistry (IHC) analysis of CD68-positive cells in the brains of rhesus macaques on 0, 1, 4, 7 and 14 dpi. Each data point represents an independent field of view from slides subjected to IHC. Arrow, positive staining. Scale bar, 100 μm. **c** Concentrations of inflammatory cytokines in the brains of rhesus macaques infected with SARS-CoV-2. **d** Concentrations of inflammatory cytokines in the serum of rhesus macaques infected with SARS-CoV-2

To determine the effects of SARS-CoV-2 post entry into the CNS, histopathological examination was performed. The entorhinal area, hippocampus, thalamus, and midbrain displayed different degrees of neuronal death, glial hyperplasia, and edema post intranasal inoculation of SARS-CoV-2 (Fig. 2a and Supplementary Tables S2 and S3). We then performed immunohistochemistry (IHC) to further identify CD68^+^ macrophage-like cells, presumably perivascular macrophages and activated microglia potentially involved in CNS inflammation. We found plenty of CD68^+^ cells infiltrating into the hippocampus and medulla oblongata (Fig. 2b). The concentrations of inflammatory cytokines, including granulocyte colony-stimulating factor (G-CSF), interleukin (IL)-2, IL-8, IL-13, IL-15, and vascular endothelial growth factor (VEGF), were elevated in the CNS tissues post intranasal virus inoculation, suggesting that the neuroinflammation was induced by SARS-CoV-2 infection (Fig. 2c). In the serum samples after infection, the increased inflammatory cytokines were IL-8 and IL-15 (Fig. 2d). Collectively, our results show that the neuroinflammation in the CNS occurs after intranasal SARS-CoV-2 inoculation.

### Neurons, astrocytes, and microglia are the potential sources of inflammatory cytokines in the CNS

To further determine the mechanism underlying SARS-CoV-2 infection in the CNS, we inoculated rhesus monkeys intracranially with a high (1 × 10^6^ plaque-forming unit (PFU)) or low (1 × 10^5^ PFU) dose of SARS-CoV-2. No significant changes of body temperature, body weight, or overt clinical signs were observed in any of the infected animals during the study (Supplementary Fig. S2a). The viral RNA became detectable in the nasal and pharyngeal swab samples on 1 dpi, in anal swabs on 2 dpi, and in blood on 5 dpi (Supplementary Fig. S2b).

**Fig. 3:**
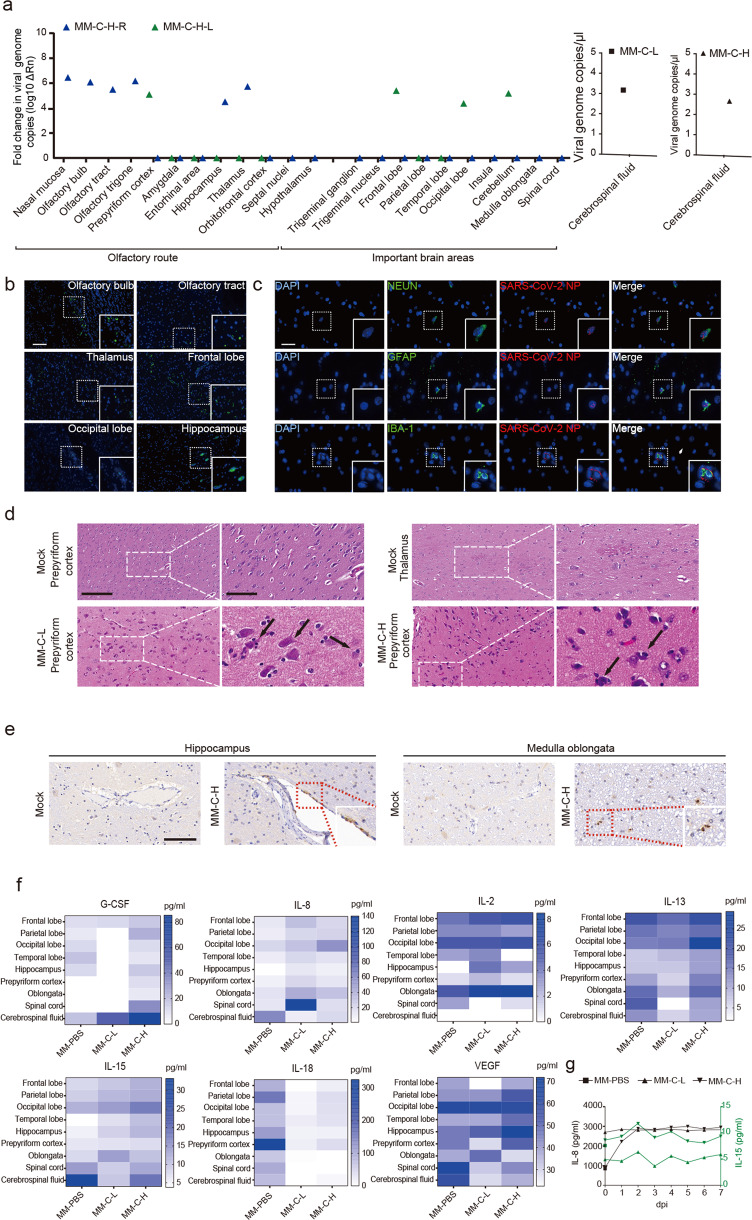
Intracranial inoculation with SARS-CoV-2 causes the neuroinflammation in multiple brain regions of rhesus monkeys. Rhesus monkeys were intracranially inoculated with a high (MM-C-H) and low (MM-C-L) dose of SARS-CoV-2. On 9 dpi, 3 animals (including PBS Control) were necropsied for the following analyses. a Viral genomic RNA in the CNS tissues (MM-C-H-L) and CSF was quantitated by qRT-PCR. **b** IF staining of N protein in the CNS tissues. Scale bar, 100 μm. The images in the solid line boxes are zoomed in from those in the dotted line boxes. **c** IF double staining for viral N protein (red) and cell surface markers (green) in thalamic infected with a high dose of SARS-CoV-2. Scale bar, 100 μm. **d** H&E staining in the brains of rhesus macaques intracranially inoculated with a high (MM-C-H) and low (MM-C-L) dose of SARS-CoV-2 or PBS. Scale bar (left panel), 50 μm; scale bar (right panel), 20 μm. e IHC for CD68-positive cells in the brains of rhesus macaques infected with a high dose of SARS-CoV-2 (MM-C-H) or PBS. Scale bar, 100 μm. **f** Concentrations of inflammatory cytokines in the brains of rhesus macaques. **g** Concentrations of inflammatory cytokines in the serum of rhesus macaques

On 9 dpi, rhesus monkeys were sacrificed for further analyses. The intracranial inoculation with high (1 × 10^6^ PFU) but not low (1 × 10^5^ PFU) dose of SARS-CoV-2 led to a widespread pattern of viruses in the CNS, including the olfactory bulb, hippocampus, thalamus, frontal lobe, and occipital lobe (Fig. 3a, left panel). The SARS-CoV-2 was not detected in the CNS tissues of the low-dose group. In the CSF, similar load of SARS-CoV-2 was observed in the monkeys inoculated intracranially with both the high and low dose (Fig. 3a, right panel). In both the groups, no obvious gross lesions were observed in the lungs (Supplementary Fig. S2c). However, hematoxylin and eosin staining of lung sections revealed a low degree of alveolar septum thickening, edema, and hemorrhage (Supplementary Fig. S2d and Tables S2 and S3).

In the CNS, viral antigen NP was intensively detected in the olfactory bulb, olfactory tract, hippocampus, thalamus, frontal lobe, and occipital lobe on day 9 after the intracranial inoculation of high dose of SARS-CoV-2 (Fig. 3b). Further double immunostaining of thalamic sections from the inoculated monkeys showed the viral antigen NP in neurons, astrocytes, and microglial cells (Fig. 3c). Similar results were also observed in the hippocampus and prepyriform cortex with intranasal inoculation (Supplementary Fig. S1d). Histopathological examination revealed various degrees of neuronal death, glial hyperplasia, edema, and a small amount of perivascular inflammatory cell infiltration in the olfactory bulb, primary olfactory cortex, and thalamus (Fig. 3d). IHC staining showed the infiltration of CD68^+^ microglia into the hippocampus and medulla oblongata (Fig. 3e). Finally, levels of G-CSF, IL-2, IL-8, IL-13, IL-15, and VEGF increased and IL-18 decreased in the CNS tissues of intracranially inoculated monkeys (Fig. 3f). Similar to that of intranasal inoculation, IL-8 in the serum increased after intracranial infection (Fig. 3g). Together, these results suggest that intracranial inoculation of SARS-CoV-2 leads to the inflammation and pathological changes by targeting some specific types of cells in the CNS.

To determine the susceptibility of cell lines derived from the CNS to SARS-CoV-2 infection, the six host factors ACE2, TMRPRSS2, ERGIC3, FUT8, LMAN2, and MGAT2 expression levels were evaluated in the CNS-derived cell lines by qRT-PCR. mRNA expression of ACE2, ERGIC3, FUT8, LMAN2, and MGAT2 were detectable in T98G, U251, SK-N-SH, normal human endothelial cell (NhEC), HA, and HMC-3 (Supplementary Fig. S3a); however, one of the key factors, TMPRSS2, was under the detectable level. Then the growth of SARS-CoV-2 in some representative cell lines was evaluated. Although viral NP could be detected by IF staining in the neuroblastoma cell line SK-N-SH, glioblastoma cell line U251, and NhECs (Supplementary Fig. S3b), viral gRNA or sgRNA showed no significant change during inoculation (Supplementary Fig. S3c). The increased levels of G-CSF, IL-8, IL-2, and VEGF were also observed in cell lines derived from neurons, glial cells, and vascular endothelium treated with SARS-CoV-2 (Supplementary Fig. S3d). These results suggested that SARS-CoV-2 could induce inflammation in the CNS without efficient replication.

### CNS cells undergo a hyperbiosynthetic, hypermetabolic, and mitochondrial disorders in response to SARS-CoV-2 infections

**Fig. 4:**
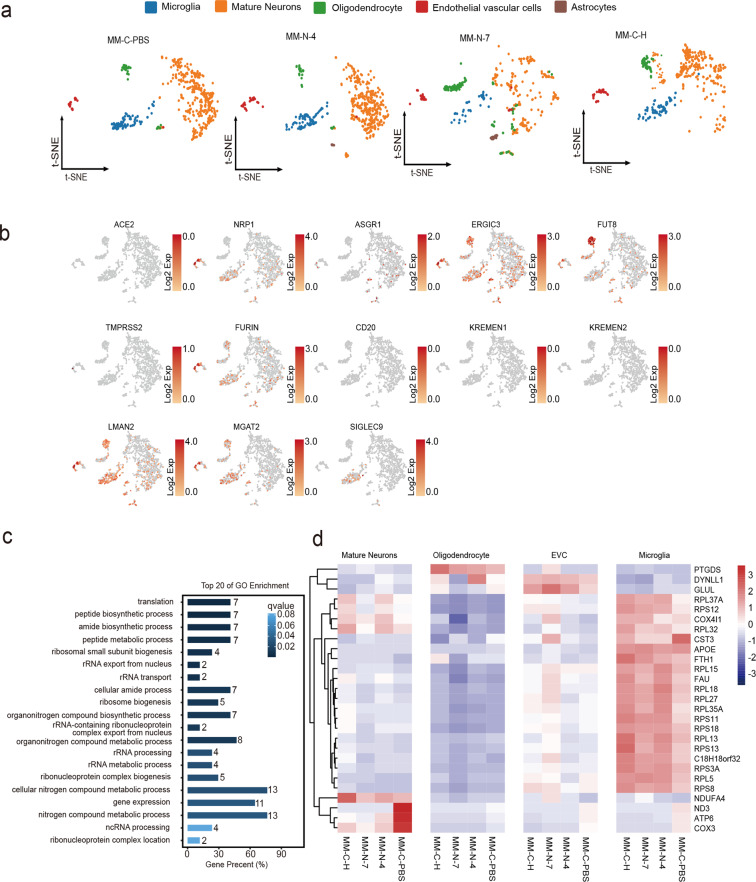
CNS cells in hippocampus undergo a hyperbiosynthetic, hypermetabolic, and mitochondrial disorders in response to SARS-CoV-2 infections. **a** t-SNE projection of cells from single-cell sequencing. **b** Heatmap of receptor genes for SARS-CoV-2 in the hippocampus of rhesus macaques. **c** Enriched GO terms in SARS-CoV-2-infected hippocampus and SARS-CoV-2 compared with control. **d** Heatmap of differentially expressed genes (DEGs) from the hippocampus infected with SARS-CoV-2 compared with control

Finally, single-cell sequencing was conducted to comprehensively determine the effects of SARS-CoV-2 infection on the CNS in consideration of the cellular heterogeneity. We analyzed the tissues collected on the 4 and 7dpi following intranasal inoculation and on the 9 dpi following intracranial inoculation. Two thousand cells were used to create five different clusters, including microglia, mature neurons, oligodendrocytes, endothelial vascular cells, and astrocytes (Fig. 4a and Supplementary Fig. S4a). Then 13 potential host factors were further analyzed in these cells of the hippocampus according to the reports by Gu et al.^24^ and Cantuti-Castelvetri et al.^20^ We found very low levels of TMPRSS2 (Fig. 4b), consistent with the results from the CNS-derived cell lines by qRT-PCR (Supplementary Fig. S3a). In the five cell types we analyzed, ERGIC3, LMAN2, and MGAT2 have relatively high levels of expression. NRP1 was expressed in microglia, mature neurons, endothelial vascular cells, and astrocytes; ASGR1 in mature neurons, endothelial vascular cells, and astrocytes; and SIGLEC9 only in microglia (Fig. 4b).

To investigate the underlying molecular mechanisms, we further examined the transcriptomic profile of SARS-CoV-2-infected tissues (Supplementary Fig. S4b). Gene ontology (GO) analysis showed that SARS-CoV-2 infection induced the enrichment of genes corresponding to translation, along with enrichment for biosynthetic processes, including peptide, amide, ribosome-related biosynthetic processes, as well as peptide, cellular amide, and nitrogen compound metabolic processes in the hippocampus (Fig. 4c and Supplementary Fig. S4c). In neurons, FTH1 and RPL15 are upregulated in the hippocampus after SARS-CoV-2 inoculation. RPS11, RPS13, and RPL13 are upregulated in the microglia of virus-inoculated hippocampus. Most of these genes are involved in biosynthetic and metabolic processes. ND3, ATP6, and COX3 involved in mitochondrial disorders were downregulated in mature neurons, oligodendrocytes, endothelial vascular cells, and microglia of hippocampus, the primary olfactory cortex, and cerebral cortex post SARS-CoV-2 inoculation (Fig. 4d and Supplementary Fig. S4d), which might hint that mitochondrial disorders were associated with the nervous system damage caused by SARS-CoV-2.

## Discussion

Patients infected with SARS-CoV-2 develop a number of neurological complications. Headache, paresthesia, anosmia, dyslexia, ataxia, and altered mental status in COVID-19 patients may be early signs of SARS-CoV-2 neurotropism.^25–27^ SARS-CoV-2 RNA was detected in the CSF of the first meningitis patient.^3^ In particular, loss of smell is considered to be a common sign of COVID-19 and can be used as one of the diagnostic indices. The mouse model expressing hACE2 shows an increased viral load in the brain after intranasal infection.^8–10^ Our recently established NHP model of COVID-19 suggests that SARS-CoV-2 could infect the CNS.^22^ In this study, we further explored the mechanism of SARS-CoV-2 invasion in the CNS on the basis of this rhesus monkey model. We found that SARS-CoV-2 transports to the CNS via the olfactory route following intranasal inoculation. Inflammatory cytokines and pathological lesions in the CNS are induced by SASR-CoV-2 infection, which potentially leads to the neurological manifestations. Indeed, we observed meningeal adhesions in some of the infected rhesus monkeys during autopsy (data not shown). Viruses in the CSF possibly accumulate in dural sinuses and induce immune responses since dural sinuses function as a neuroimmune interface in the CNS.^28^ We also observed a trend of increased moving distance and velocity, which may related to nervous system damage (data not shown). However, due to the limitations of the P4 laboratory on behavioral research, the data are very rough, and we did not include it in the article.

In lethal COVID-19 patients, their behaviors and postmortem exams indicate that SARS-CoV-2 could enter the CNS and cause neurological diseases possibly by crossing the neural–mucosal interface in olfactory mucosa, which is evidenced by the pathological changes and the existence of viral particles in the CNS.^7^ However, the relatively long postmortem interval (even >3 days) in these clinical studies makes it hard to precisely explain the pathological changes due to the autolysis of cells and tissues. Therefore, these conclusions need to be confirmed in SARS-CoV-2 infection models. There are two kinds of models currently utilized for evaluation of SARS-CoV-2 infection in the CNS. The first model is SARS-CoV-2 infection of human induced pluripotent stem cell-derived brain organoids in vitro, which reveals the SARS-CoV-2 infection of neuronal cells in vitro and is ACE2-dependent.^9,29^ The second is SARS-CoV-2 infection of mice transduced by a hACE2-expressing adeno-associated virus vector. In this model, intranasal inoculation of SARS-CoV-2 results in the CNS infection, but no viral antigen was detected in astrocytes or microglia cells. The K18-hACE2 mice demonstrate that SARS-CoV-2 neuroinvasion and encephalitis is associated with mortality in these mice.^10^ No rapid axonal transport of SARS-CoV-2 to the brain has been demonstrated in the hamster model during the first 2 weeks after infection.^30^ In our present study, we observed SARS-CoV-2 protein not only in neurons but also in astrocytes and microglia of rhesus monkeys. However, there are only a very small number of positive cells (Fig. 3c). Even though the hACE2 mice models confirmed the SARS-CoV-2 infection of the CNS, the distribution of hACE2 in mice cannot recapitulate the real situation of human natural infection well. One of the main arguments against SARS-CoV-2 neuroinvasion lies in the fact that the mRNA levels of ACE2 appear to be very low in the CNS, which is confirmed by the single-cell sequencing results in this study (Fig. 4b). In fact, several recent studies report some new SARS-CoV-2-binding receptors, including neuroreceptors,^20,24,31^ suggesting that SARS-CoV-2 may use other receptors for neurotropism in addition to ACE2. Our data show that the virus cannot replicate effectively in the CNS, so we speculate that SARS-CoV-2 causes pathological damage to the CNS through indirect factors such as “cytokine storms”.

SARS-CoV-2 invasion of the CNS is consistent with the neurotropic organotropism of other CoVs, such as SARS-CoV^12^ and Middle East respiratory syndrome-CoV.^13^ Our results suggest that SARS-CoV-2 primarily enters the CNS via the olfactory nerve and transports quickly to the brain. Olfactory bulb infection occurred as early as 1 dpi (Fig. 1b). Clinically, SARS-CoV-2 was detected in the olfactory nerve and hypothalamus of COVID-19 patients.^32^

In this study, we also found SARS-CoV-2 viral protein in hippocampus, thalamus, entorhinal area, and medulla oblongata post intranasal inoculation (Fig. 1c). Infection of the cardiorespiratory center in the medulla oblongata could contribute to death in severe cases.^33,34^ Localization of SARS-CoV-2 in the hippocampus suggests that evaluation of memory should be recommended in COVID-19 patients. In contrast to our observation, viral RNA could not be detected in the brain stem on days 3, 4, or 21 post inoculation in another two NHP model of SARS-CoV-2 infection.^23,35^ The difference between this model and ours can be explained probably by the intranasal administration of different doses of SARS-CoV-2.

Consistent with the study that examined viral protein in brain sections from SARS-CoV-2-infected hACE2 knock-in mice,^8^ we have detected the viral NP expression in neurons, astrocytes, and microglial cells of rhesus monkeys (Fig. 3c), but in vitro no viral replication was observed in the CNS-associated cell lines (Supplementary Fig. S3c).

Also, we did a lot of ultra-thin sections of electron microscopy, but unfortunately, we did not find the viral particles in the brain tissue sections. The reason may be that the virus replication is limited in CNS, and the field of the electron microscopy is also limited on the location of virus particles. These suggest that SARS-CoV-2 could invade the CNS but not infect and replicate efficiently. These CNS-associated cell lines are less susceptible to SARS-CoV-2 infection, which was previously reported,^36^ probably due to lack of TMPRSS2 (Supplementary Fig. S3a). However, we did observe some pathological damage (neuronal death, glial hyperplasia, and edema) in the CNS (Figs. 2a and 3d), which may be caused by the elevated inflammatory cytokines in the serum or CNS tissues (Figs. 2c and 3f). As in the lung of COVID-19, excessive levels of proinflammatory cytokines/chemokines might result in a “cytokine storm” that causes injury in the CNS.^37^ Virus invasion of the CNS leads to the induction of neuroinflammatory CNS disorder-related cytokines/chemokines, such as G-CSF,^38^ IL-8,^39^ IL-13,^40^ and IL-15.^41^ IL-15 is an immunoregulatory cytokine with antiviral properties.^42^ The elevated level of IL-15 observed in the infected CNS might be due to the compensatory anti-inflammatory response of IL-15 to severe diseases (Fig. 2d). High levels of G-CSF, IL-2, and IL-8 were observed in the infected CNS, which might be one of the reasons for the deterioration of the patient’s condition.^19,43^

The increasing trend of IL-15 concentration after intracranial injection is consistent with that observed in the brain and blood after intranasal inoculation, indicating that IL-15 may be a clinical sign of brain damage. IL-18 is a cytokine that protects against viral infection and has been reported to be upregulated in COVID-19 patients during the recovery period.^44^ In all the analyzed CNS tissues except the temporal lobe, we found a decrease in the level of IL-18 on 7 dpi. The single-cell sequencing results indicated that the inflammatory cytokines induced by SARS-CoV-2 infection have downregulated some mitochondria-associated genes (Fig. 4d and Supplementary Fig. S4d). Mitochondrial disorders may cause Alzheimer’s disease,^45^ which provides some hints for the prognosis of COVID-19 patients.

There are three potential routes for SARS-CoV-2 to reach the CNS.^7,26^ The results in this study suggest that the olfactory route is involved in the transport of SARS-CoV-2 to the CNS. In addition, we found viral RNA in the blood and in the CSF on day 1 after nasal infection but not for every animal (Fig. 1b and Supplementary Fig. S1b). The possibility of lymphatic/hematogenous spread of SARS-CoV-2 cannot be ruled out, especially in the late stage of infection. In the hematogenous route, the virus may infect endothelial cells of the blood–brain barrier or leukocytes to disseminate into the CNS.^46^ ACE2 is widely expressed on the epithelial cells of the oral mucosa.^47^ Moreover, the olfactory route is a shortcut for many viruses to enter the CNS.^48^ Olfactory receptor neurons project dendrites into the nasal cavity and extend axons through the cribriform plate into the olfactory bulb.^48^ Meanwhile, hyposmia and hypogeusia are reported in some COVID-19 patients.^49^ These evidences could partly support our conclusion. Published work by our team suggests that gastrointestinal tract is an alternative route for SARS-CoV-2 infection in rhesus monkeys,^50^ suggesting that enteric nervous system might be an alternative route for SARS-CoV-2 infection in the CNS, a possibility that deserves further study in the future.

The in vitro organoid model and ACE2-transgenic murine model showed the severe infection of SARS-CoV-2 in the CNS. However, the physiological expression and distribution of ACE2 in human beings is distinctly different from these reported models. In this study, rhesus monkeys, genetically close to human beings, were used as a model to simulate the SARS-CoV-2 infection in human beings. Therefore, this model can recapitulate the SARS-CoV-2-induced damages in the CNS of human beings. Inflammatory cytokines induced by SARS-CoV-2 infection but not viral replication play important roles in the CNS diseases. Overall, the findings in this study indicate that SARS-CoV-2 invades the CNS via olfactory route in rhesus monkeys following intranasal inoculation. The drawbacks of this study are only one animal for each group and the limited techniques. Therefore, more animals and the multiomics approach for further research are essential to elucidate the host and viral mechanisms involved in SARS-CoV-2-induced damage to the CNS, which will provide important insights into the CNS-related pathogenesis of SARS-CoV-2.

## Materials and methods

### Animals and experimental procedures

Nine rhesus monkeys (3–5 kg, 3–5 years old) were used for this study (Table S1). Five monkeys were intranasally infected with 1 × 10^7^ PFU of SARS-CoV-2 in 1 mL of PBS, and two monkeys were intracranially injected with 1 × 10^6^ or 1 × 10^5^ PFU of SARS-CoV-2 in 200 µL of PBS. One monkey was intranasally and intracranially treated with PBS as a control. One monkey was used as an untreated control without any treatment. These two control monkeys were shared with the other experiment to follow the 3R principle. The clinical signs of the monkeys, including body temperature and body weight, were checked daily after infection and sample collection, and necropsies were performed according to standard experimental procedures (Fig. 1a).

Rhesus monkeys were bred and provided by the Kunming Primate Center of the Chinese Academy of Medical Sciences (Laboratory animal production license #SCXK (Dian)-K2015-0004). The monkeys were lightly anesthetized with ketamine (6 mg/kg) before the experimental operation. All animal procedures were approved by the Institutional Animal Care and Use Committee of Institute of Medical Biology, Chinese Academy of Medical Science (ethics number: DWSP202002001) and performed in the high-level biosafety containment facility of National Kunming High-Level Biosafety Primate Research Center (Yunnan, China).

### Cell culture and virus infection

SARS-CoV-2 viral stock was kindly provided by the Guangdong Provincial Center for Disease Control and Prevention. SARS-CoV-2 was propagated in Vero-E6 cells and qualitatively detected by RT-PCR, sequencing, and transmission electronic microscopy and quantitatively measured by the plaque assay.

T98G, U251, and HMC-3 cells were maintained in minimum Eagle’s medium supplemented with 10% fetal bovine serum (FBS), penicillin and streptomycin, 1% non-essential amino acid (NEAA), and 2% sodium pyruvate. SK-N-SH cells were maintained in Dulbecco’s Modified Eagle’s Medium (DMEM) supplemented with 10% FBS, penicillin and streptomycin (10,000 IU/mL), 1% NEAA, and 2% sodium pyruvate. SHSY5Y cells were maintained in Roswell Park Memorial Institute 1640 (RPMI1640) medium supplemented with 10% FBS, penicillin and streptomycin (10,000 IU/mL). HA and HA-sp cells were maintained in astrocyte medium supplemented with 2% FBS, 1% astrocyte growth supplement, and penicillin and streptomycin (10,000 IU/mL). NhECs and human umbilical vein endothelial cells were maintained in EBM2 basal medium supplemented with EGM2 supplement and penicillin and streptomycin (10,000 IU/mL). All these cell lines were cultured at 37 °C in a humidified CO_2_ incubator.

Cells were incubated with SARS-CoV-2 at a multiplicity of infection of 0.5 for 30 min at 37 °C and washed for three times with PBS. Then we maintained cells in DMEM supplemented with 2% FBS and penicillin and streptomycin (10,000 IU/mL). At 0, 2, 8, 12, 24, 36, 48 and 72 h post inoculation with viruses, cell culture was harvested for viral RNA quantitation by qRT-PCR.

All work related to infectious SARS-CoV-2 was performed in the high-level biosafety facility of the National Kunming High-Level Biosafety Primate Research Center (Yunnan, China).

### qRT-PCR for viral genomic RNA and host genes

Viral genomic RNA was quantitated as previously described.^22^ Briefly, a Direct-zol RNA Mini Prep Extraction Kit was used to isolate total RNA, and reverse transcription was performed with TaqMan Fast Virus 1-Step Master Mix to obtain cDNA. qRT-PCR was performed by using TaqMan® Universal PCR Master Mix, the CFX384 Touch Real-time PCR detection system (Bio-Rad, USA), and the Applied Biosystem 7500 system. N gene cDNA primers and probes were synthesized based on the sequences reported by the Chinese Center for Disease Control and Prevention. The sequences of N gene cDNA primers and probes are as follows: forward/leader primer: GGGGAACTT-CTCCTGCTAGAAT, reverse primer: CAGACATTTTGCTCTCAAGCTG, and probe: 5’-FAM TTGCTGCTGCTTGACAGATT-TAMRA-3’.

Primers and probes for the ACE2 gene were synthesized based on the NM_001135696.1 sequence in GenBank. The sequences are as follows: forward primer: GAACCCTGGACCCTAGCATT, reverse primer: TTCTGGTCTTTCAGCCAGGT, and probe: 5′-FAM ACCACTGCTCAACTACTTTGAGCCCC-TAMRA-3′. Primers and probes for the TMPRSS2 gene were synthesized based on the XM_028845315.1 sequence in GenBank. The sequences are as follows: forward primer: AAGGGAAGACCTCAGACGTG, reverse primer: CACAGATCATGGCTGGTGTG, and probe: 5′-FAM CCGCGGCTCAATGAGAGGCAGGA-CCTA-TAMRA-3′. Primers and probes for the ERGIC3 gene were synthesized based on the NM_015966.3 sequence in GenBank. The sequences are as follows: forward primer: TTGTC-AGTGGCCTTCTCATG, reverse primer: ATCTTCAGTTTATCTCCCCGC, and probe: 5’-FAM CTGTTCCTGTCCGAGCTGCAGTAT-TAMRA-3’. Primers and probes for the FUT8 gene were synthesized based on the NM_001371533.1 sequence in GenBank. The sequences are as follows: forward primer: TTCAGAATCCCAAGGACTGC, reverse primer: TCCAAG-ATGAGTGTTCGCTG, and probe: 5’-FAM TGACAGCCATAGCCACAGCCTT-TAMRA-3’. Primers and probes for LMAN2 gene were synthesized based on the NM_006816.3 sequence in GenBank. The sequences are as follows: forward primer: AGATAACTTCCAC-GGCTTAGC, reverse primer: CATCCTTGCTGTGGTCGTAG, and probe: 5’-FAM TCTCGGTGATGGTG-AACAATGGCTC-TAMRA-3’. Primers and probes for the MGAT2 gene were synthesized based on the NM_002408.4 sequence in GenBank. The sequences are as follows: forward primer: CTGAGGAATGTAGATAAGGCTGG, reverse primer: ATGGCTAAAGAT-GACGAGGAC, and probe: 5’-FAM TTCCCTGGGCTTTTCGAA-GTGAGTCTAMRA-3’. One-step RT-PCR was performed under the following conditions: 25 °C for 2 min, 50 °C for 15 min, and 95 °C for 2 min followed by 40 cycles at 95 °C for 5 s and 60 °C for 31 s.

### Histology and IHC analysis

Histological exams and IHC were performed according to the manufacture-recommended procedures. Rabbit anti-ACE2 and anti-CD68 primary antibodies were used for IHC. Positive staining was detected with diaminobenzidine. IHC sections were evaluated with a blinded manner.

The tissue was fixed with formalin to prepare 5-μm paraffin sections. The dewaxed and hydrated sections were heated to boiling with 10 mM citrate buffer (pH 6.0) for 20 min. The sections were treated with 3% H_2_O_2_ at room temperature for 10 min to block endogenous peroxidase activity and then incubated with primary antibody overnight at 4 °C.

### IF assay

IF was performed according to the manufacture-recommended procedure. Rabbit anti-N protein (1:500, kindly provided by Professor Zhengli Shi’s group), anti-NeuN, anti-GFAP, and anti-IBA-1 were used as primary antibodies for IF analysis. Images were captured under a fluorescence microscope (Leica).

Cells were fixed on slides with 4% paraformaldehyde at room temperature for 20 min and permeabilized with 0.5% Triton X-100 for 2 min. The cells were incubated with primary antibody overnight, washed three times with PBS, incubated with the corresponding fluorescent secondary antibody for 30 min, and stained with DAPI.

### Multiplex cytokine assay

The MILLIPLEX MAP Non-Human Primate Cytokine Magnetic Bead Panel-Immunology Multiplex Assay was conducted to analyze multiple cytokines in serum, including IL-1β, IL-4, IL-5, IL-6, IL-8/CXCL8, G-CSF, GM-CSF, IFN-γ, IL-1rα, IL-2, IL-10, IL12p40, IL-13, IL-15, IL-17A/CTLA8, MCP-1/CCL2, MIP-1β/CCL4, MIP-1α/CCL3, sCD40L, TGF-α, TNF-α, VEGF, and IL-18, according to the manufacturer’s instructions.

Bio-Plex Pro Human Cytokine Screening Panel, 48-Plex Kit was conducted to analyze multiple cytokines in cell culture-medium, including IL-1β, IL-4, IL-5, IL-6, IL-8/CXCL8, G-CSF, GM-CSF, IFN-γ, IL-1rα, IL-2, IL-10, IL12p40, IL12p70, IL-13, IL-15, IL-17A/CTLA8, MCP-1/CCL2, MIP-1β/CCL4, MIP-1α/CCL3, TNF-α, VEGF, IL-18, FGF basic, Eotaxin, IL-2rα, IL-3, IL-16, IL-7, IL-9, GRO-α, HGF, IFN-α2, LIF, MCP-3,IP-10, MIG, β-NGF, SCF, SCGF-β, SDF-1α, PDGF-BB, RANTES, CTACK, MIF, TRAIL, M-CSF, and TNF-β, according to the manufacturer’s instructions.

### Single-cell RNA-sequencing (scRNA-seq) analysis

Single-cell suspensions were made using a gentle MACS Octo (Miltenyi Biotec). Single-cell suspensions were loaded onto the Chromium Controller (10× Genomics) for droplet formation. scRNA-seq libraries were prepared using the Chromium Single Cell 3′ Reagent Kit (10× Genomics). The cell suspension (300–600 living cells/mL) was loaded onto the Chromium single-cell controller (10× Genomics) by using the single-cell 3’ Library and Gel Bead Kit V3 (10× Genomics, 1000075) and Chromium Single Cell B Chip Kit (10× Genomics, 1000074). Details are shown in the Supplementary File. scRNA-seq libraries were constructed by the Single Cell 3’ Library and Gel Bead Kit V3. The libraries were sequenced by an Illumina Novaseq6000 sequencer, the sequencing depth of each cell was at least 100,000 reads, and the paired-end 150 bp (PE150) reading strategy (CapitalBio Technology, Beijing) was adopted. The Cell Ranger software was obtained from 10× Genomics website. Alignment, filtering, barcode counting, and UMI counting were performed with cell ranger count module. Dimensionality reduction and the first ten principal components were used to generate clusters by *K*-means algorithm and graph-based algorithm, respectively. Details of other analyses are shown in the Supplementary Files.

#### Cell capture and cDNA synthesis

For cell capture and cDNA synthesis, single cells were suspended in PBS containing 0.04% bovine serum albumin. About 8000 cells were added to each channel, and it is estimated that about 2200 target cells will be recovered. The captured cells were lysed and the released RNA was barcoded in a single GEM by reverse transcription. Reverse transcription was performed on the S1000TM Touch Thermal Cycler (Bio Rad) at 53 °C for 45 min, then at 85 °C for 5 min and held at 4 °C. The quality of cDNA was evaluated using Agilent 4200 (CapitalBio Technology, Beijing).

#### Data preprocessing using Seurat pipeline

Another clustering method is Seurat 3.0 (R package). Cells with <200 genes, or the top 1% of genes, or >25% of mitochondrial genes, are considered abnormal and filtered out. Dimensionality reduction was performed by principal component analysis, and visualization was realized by TSNE and UMAP.

#### Enrichment analysis

GO enrichment, KEGG enrichment, Reactome enrichment, and Disease enrichment (human only) of cluster markers were performed using the KOBAS software. The results were visualized using R package.

#### Protein–protein interaction (PPI)

PPI was obtained from STRING database with combine score ≥400. For each cluster, the interaction of top 20 marker genes was selected from the database. The results were visualized using the Cytoscape software.

#### Transcription factor prediction

Transcription factors were predicted within 2000 bp upstream and 500 bp downstream of transcription start site for top 20 marker genes of each cluster using TFBS Tools and JASPAR database. The gene and TF network were visualized using the Cytoscape software.

#### Gene Set Enrichment Analysis (GSEA) assay

GSEA was performed by using the GSEA software (version 2.2.2.4). Gene expression data was calculated by the average umi count of genes in one cluster and the remaining clusters, respectively. The minimum and maximum criteria for selecting a gene set from the collection are 0 and 500 genes.

#### Single-cell trajectory analysis

Single-cell trajectories were built with Monocle (R package) that introduced pseudo time. Genes were filtered by the following criteria: expressed in >10 cells, the average expression value was >0.1, Qval was <0.01 in different analysis.

#### Cell cycle phase

Cell cycle phase was assigned by Seurat 3.0.

#### Weighted correlation network analysis (WGCNA)

WGCNA was performed by The WGCNA R software package. Each cluster was divided into subclusters, and the average expression of gene in a subcluster was calculated.

#### Cell type annotation

Cell types were annotated by single R (https://bioconductor.org/packages/devel/bioc/html/SingleR.html). By using the reference transcriptome data set of pure cell types to independently infer the cell of origin of each single cell, unbiased cell-type identification from scRNA-seq data was performed.

## Supplementary information

Supplementary Materials

## Data Availability

The accession number for the single-cell RNA-seq data reported in this study is GSE167319 in GEO.
