# Supplementary material for: The olfactory route is a potential way for SARS-CoV-2 to invade the central nervous system of rhesus monkeys
Source: Signal Transduct Target Ther. 2021 Apr 24;6:169. doi: 10.1038/s41392-021-00591-7 (PMC8065334; doi:10.1038/s41392-021-00591-7)
Supplement: Supplementary file 1 — Supplementary Materials [file 41392_2021_591_MOESM1_ESM.docx]

Supplementary Materials for

**The Olfactory Route isa Potential Way forSARS-CoV-2 to Invade the Central Nervous System of Rhesus Monkeys**

Authors:Li Jiao^1#^, Yun Yang^1#^, Wenhai Yu^1#^, Yuan Zhao^1#^,Haiting Long^1^, Jiahong Gao^1^, Kaiyun Ding^1^, Chunxia Ma^1^, Jingmei Li^1^, Siwen Zhao^1^, Haixuan Wang^1^, Haiyan Li^1^, Mengli Yang^1^, Jingwen Xu^1^, Junbin Wang^1^, Jing Yang^1^, Dexuan Kuang^1^, Fangyu Luo^1^, Xingli Qian^1^, Longjiang Xu^1^, Bin Yin^2^, Wei Liu^3^,Hongqi Liu^1^*, Shuaiyao Lu^1,2^*，Xiaozhong Peng^1,2^*

Correspondence to:[pengxiaozhong@pumc.edu.cn](mailto:pengxiaozhong@pumc.edu.cn);Email: [lushuaiyao-km@163.com](mailto:lushuaiyao-km@163.com); lhq@imbcams.com.cn;

**This PDF file includes:**

Fig. S1 to S4

Tables S1 to S3


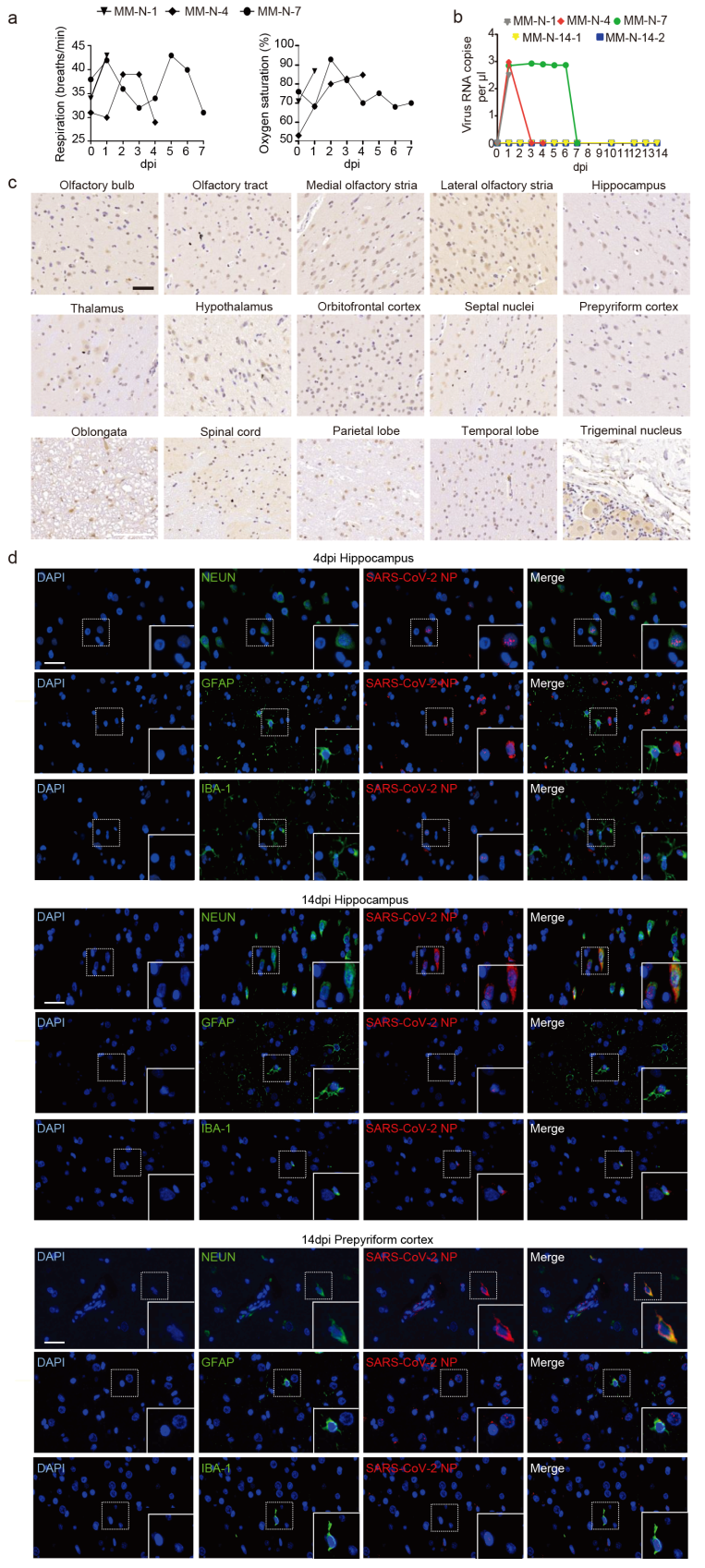


**Fig.S1.** Rhesus monkeys (3-5 years old) were intranasally inoculated with 1x 10^7^ PFU of SARS-CoV-2 in 1 ml PBS. (a)Changes in respiration and oxygen saturation inrhesus macaquesintranasally infected with SARS-CoV-2 between 0 and 7days postinoculation (dpi).(b)Viral load in the blood of SARS-CoV-2-infected rhesus macaques collected on 1, 4, 7 and 14 dpi.(c)Immunochemistry (IHC)forACE2-positive cells in the brain tissues of the uninfected rhesus macaque. Each panel represents an independent field of view from the slides subjected to IHC.Scale bar, 50μm.(d)IF double staining for viral N protein (red) and cell surface markers (green) in hippocampus and prepyriformpost inoculation with a high dose of SARS-CoV-2. Scale bar, 100 μm.

**
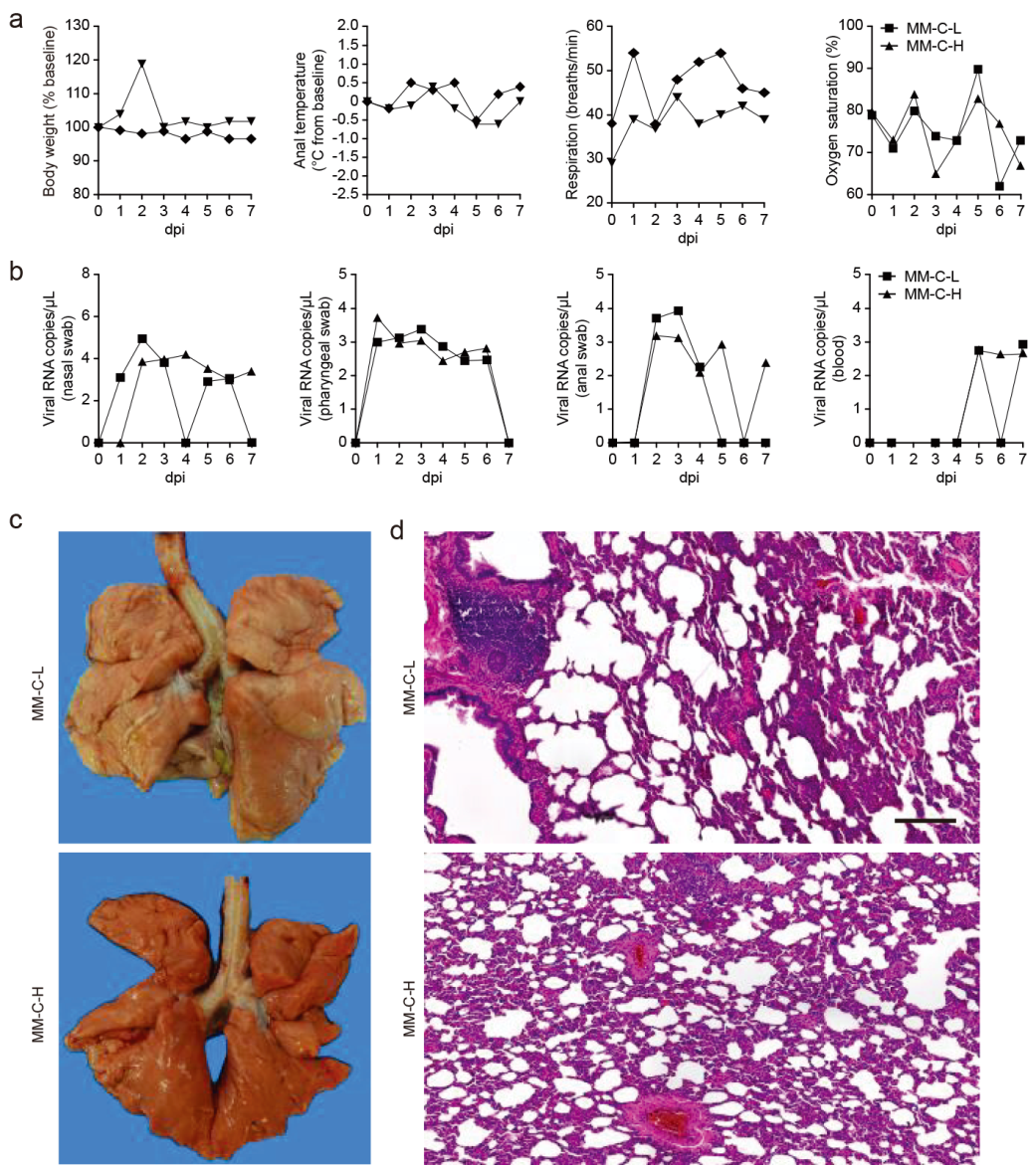
**

**Fig.S2.**Rhesus monkeyswere intracranially inoculated with a high (MM-C-H) or low (MM-C-L) dose of SARS-CoV-2. (a)Changes in bodyweight, anal temperature, respiration and oxygen saturation between 0 and 7 dpi. (b) Viral load in the nose, anus, throat, and blood was evaluatedby quantitative real-time polymerase chain reaction (qRT-PCR).(c) Gross lesion in the lungs of SARS-CoV-2-infected monkeys.(d)Histopathological analysis of hematoxylin and eosin (H&E)-stained lung tissues after intracranial inoculation. Each panel represents an independent field of view from H&E-stained slides.Scale bar, 50μm.

**
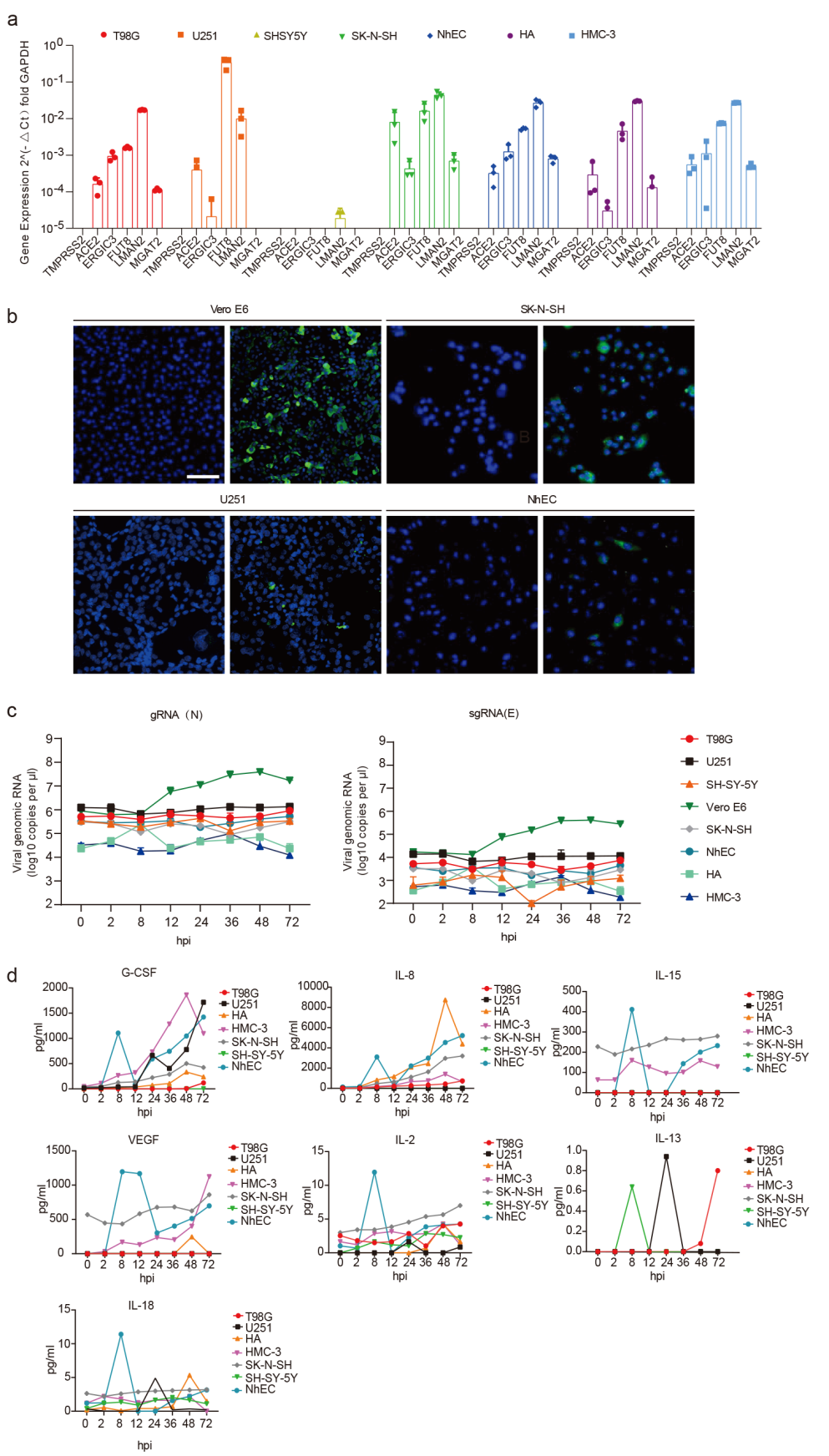
**

**Fig.S3.**(a)qRT-PCR analysis of ACE2, TMRPRSS2, ERGIC3,FUT8,LMAN2 and MGAT2 mRNA expression in uninfected CNS cell lines.(b) IF staining of viral proteininthe CNS-derived SH-N-SH cells, U87MG cells, NhECs and Vero E6 cellsinfected with SARS-CoV-2. Cell was infected with SARS-CoV-2 at the MOI of 0.5. At 24 hours post infection, cells were fixed with 4% paraformaldehyde for immunofluorescent staining with anti-NP antibody (green) and DAPI (blue). Scale bar, 100 μm.(c) (c)One-step growth curves in the culture medium of cell lines incubation with SARS-CoV-2.(d)Concentrations of inflammatory cytokines in the culture medium of cell lines.

**
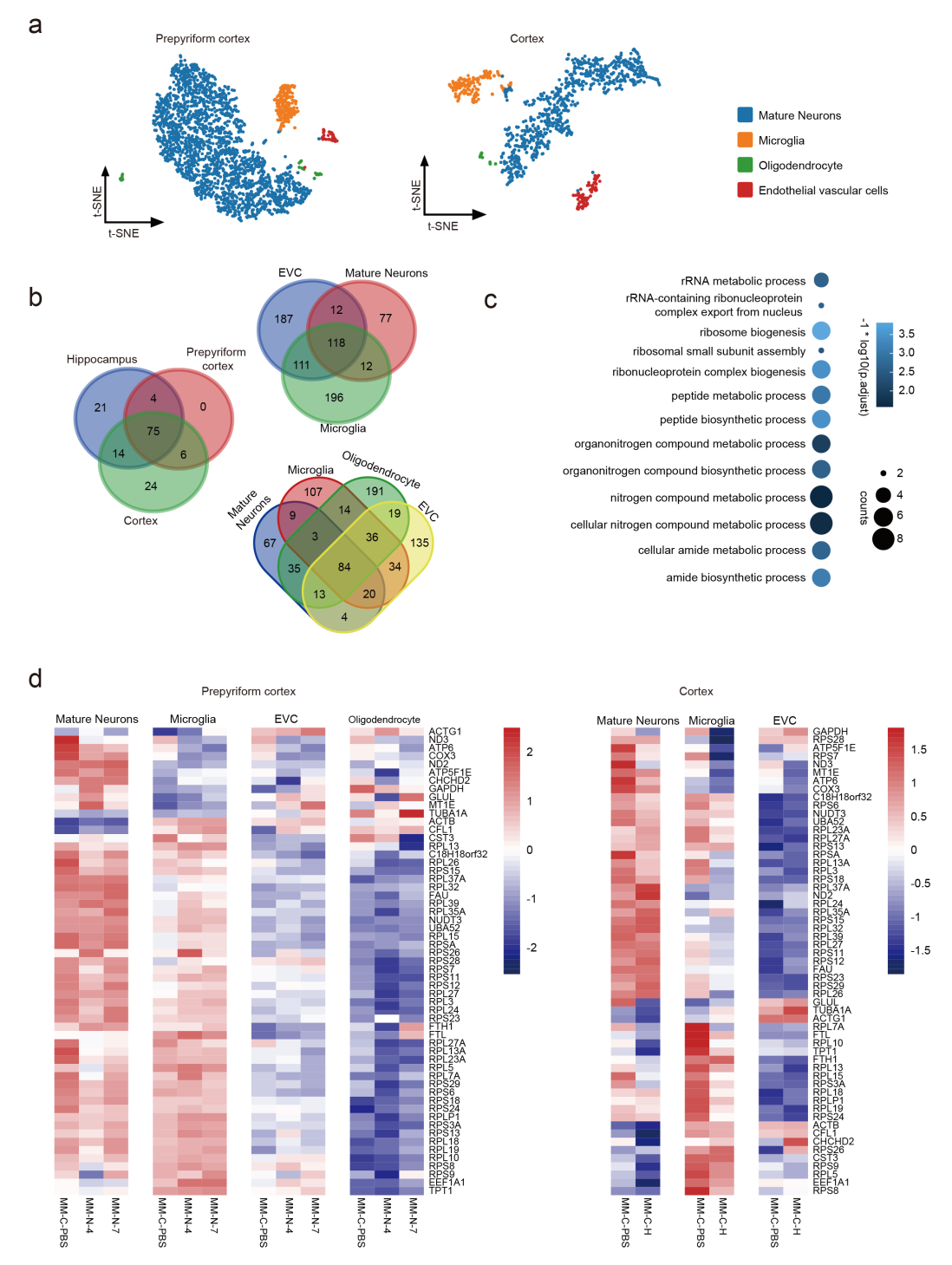
**

**Fig.S4.** (a) t-SNE projection of cells from single cell sequencing. (b) Venn diagram of four clusters significant genes. (c) Enriched GO terms in SARS-CoV-2 infected the primary olfactory cortexand cerebral cortex compared with control.(d)Heatmap of differentially expressed genes (DEGs) from the primary olfactory cortex and cerebral cortex infected with SARS-CoV-2 compared with control.

Table S1.

| **Animal information** | | | | |  | |
| --- | --- | --- | --- | --- | --- | --- |
|  | | | | |  | |
| **Animal** | **Gender** | **Treatment** | **dosage(pfu)** | **Days for observation** | |  |
| MM-N-0 | M | Intranasally | 1 ml PBS | 14^*^ | |  |
| MM-N-1^#^ | M | Intranasally | 10^7 | 1 | |  |
| MM-N-4 | M | Intranasally | 10^7 | 4 | |  |
| MM-N-7 | M | Intranasally | 10^7 | 7 | |  |
| MM-N-14-1 | M | Intranasally | 10^7 | 14 | |  |
| MM-N-14-2 | M | Intranasally | 10^7 | 14 | |  |
| MM-C-PBS^§^ | M | Intracranially | 200ulPBS | 9 | |  |
| MM-C-L | M | Intracranially | 10^5 | 9 | |  |
| MM-C-H | M | Intracranially | 10^6 | 9 | |  |
|  | | | | | |  |
| ^#^ The letter "N" represents the intranasal route. | | | |  | |  |
| ^§^The letter "C" represents the intracranial route. | | | |  | |  |
| ^*^ The number "1, 4, 7, 9, 14" represents the period of observation (day). | | | | | |  |

Table S2.

. Histopathological grade criteria

| Marker | Feature graded | Grade | Description |
| --- | --- | --- | --- |
| A | Dead neurons | + | Normal/absent |
|  |  | ++ | Rare |
|  |  | +++ | Multiple, uncommon |
|  |  | ++++ | Multiple, common, coalescing lesions seen in most fields |
| B | Glial hyperplasia and edema | + | Normal/absent |
|  |  | ++ | Rare |
|  |  | +++ | Multiple, uncommon |
|  |  | ++++ | Multiple, common, coalescing lesions seen in most fields |
| C | Lymphocytic perivascular cuffing | + | Normal/absent |
|  |  | ++ | Rare |
|  |  | +++ | Multiple, uncommon |
|  |  | ++++ | Multiple, common, coalescing lesions seen in most fields |

Table S3.

**.**

Histopathological evaluation of the brain tissues of rhesus monkeysinfected with SARS-CoV-2

| Inoculation |  | Intranasal | | |  | Intracranial | | |
| --- | --- | --- | --- | --- | --- | --- | --- | --- |
|  |  | 10^7^ PFU | 10^7^ PFU | 10^7^ PFU |  | 200 µl PBS | 10^5^ PFU | 10^6^ PFU |
| Animal |  | MM-N-1 | MM-N-4 | MM-N-7 |  | MM-C-PBS | MM-C-L | MM-C-H |
| Olfactory bulb |  | *A^+^,B^+^,C^+^ | A^+^,B^+^,C^+^ | A^+^,B^+^,C^+^ |  | A^+^,B^+^,C^+^ | A^+++^,B^++++^,C^+^ | A^+^,B^+^,C^+^ |
| Olfactory tract |  | -,-,- | -,-,- | -,-,- |  | A^+^,B^+^,C^+^ | A^+^,B^+^,C^+^ | A^+^,B^+^,C^+^ |
| Olfactory trigone |  | -,-,- | -,-,- | -,-,- |  | A^+^,B^+^,C^+^ | A^+^,B^+^,C^+^ | A^+^,B^+^,C^+^ |
| Prepyriform cortex |  | -,-,- | -,-,- | -,-,- |  | A^+^,B^+^,C^+^ | A^++^,B^++^,C^+^ | A^+^,B^++^,C^+^ |
| Entorhinal area |  | A^+^,B^+^,C^+^ | A^++^,B^+^,C^+^ | A^+++^,B^+++^,C^+^ |  | A^+^,B^+^,C^+^ | A^+^,B^+^,C^+^ | A^+^,B^+^,C^+^ |
| Hippocampus |  | A^+^,B^+^,C^+^ | A^++^,B^++^,C^+^ | A^+^,B^+^,C^+^ |  | A^+^,B^+^,C^+^ | A^+^,B^+^,C^+^ | A^+^,B^+^,C^+^ |
| Thalamus |  | A^+^,B^+^,C^+^ | A^++^,B^++^,C^++^ | A^+^,B^+^,C^+^ |  | A^+^,B^+^,C^+^ | A^++^,B^++^,C^+++^ | A^+^,B^+^,C^+^ |
| Parietal lobe |  | -,-,- | -,-,- | -,-,- |  | A^+^,B^+^,C^++^ | A^+^,B^+^,C^+^ | A^+^,B^+^,C^+^ |
| Occipital lobe |  | -,-,- | -,-,- | -,-,- |  | A^+^,B^+^,C^+^ | A^+^,B^+^,C^+^ | A^+^,B^+^,C^+^ |
| Cerebellum |  | -,-,- | -,-,- | -,-,- |  | A^+^,B^+^,C^+^ | A^+^,B^+^,C^+^ | A^+^,B^+^,C^+^ |

* The markers A, B and C are described in the supplementary Table 2 of histopathological grade criteria.
